# A fundamental catalytic difference between zinc and manganese dependent enzymes revealed in a bacterial isatin hydrolase

**DOI:** 10.1038/s41598-018-31259-y

**Published:** 2018-08-30

**Authors:** Theis Sommer, Kaare Bjerregaard-Andersen, Lalita Uribe, Michael Etzerodt, Gregor Diezemann, Jürgen Gauss, Michele Cascella, J. Preben Morth

**Affiliations:** 1 Norwegian Center for Molecular Medicine, Nordic EMBL Partnership University of Oslo, Gaustadalléen 21, 0349 Oslo, Norway; 2 Department of Chemistry, University of Oslo, Sem Sælands vei 26, 0371 Oslo, Norway; 3 Institute for Experimental Medical Research, Oslo University Hospital, 0424 Oslo, Norway; 4 Department of Molecular Biology and Genetics, Aarhus University, Gustav Wieds vej 10C, 8000 Aarhus, Denmark; 5 Institut für Physikalische Chemie, Johannes Gutenberg-Universität Mainz, Duesbergweg 10-14, 55128 Mainz, Germany; 6 Graduate School Materials Science in Mainz, Johannes Gutenberg-Universität Mainz, Staudinger Weg 9, 55128 Mainz, Germany; 7 Hylleraas Centre for Quantum Molecular Sciences, University of Oslo, Sem Saelands vei 26, 0371 Oslo, Norway; 8 Enzyme and Protein Chemistry, Section for Protein Chemistry and Enzyme Technology, Department of Biotechnology and Biomedicine, Technical University of Denmark, Søltofts Plads, 2800 Kgs. Lyngby, Denmark

## Abstract

The catalytic mechanism of the cyclic amidohydrolase isatin hydrolase depends on a catalytically active manganese in the substrate-binding pocket. The Mn^2+^ ion is bound by a motif also present in other metal dependent hydrolases like the bacterial kynurenine formamidase. The crystal structures of the isatin hydrolases from *Labrenzia aggregata* and *Ralstonia solanacearum* combined with activity assays allow for the identification of key determinants specific for the reaction mechanism. Active site residues central to the hydrolytic mechanism include a novel catalytic triad Asp-His-His supported by structural comparison and hybrid quantum mechanics/classical mechanics simulations. A hydrolytic mechanism for a Mn^2+^ dependent amidohydrolases that disfavour Zn^2+^ as the primary catalytically active site metal proposed here is supported by these likely cases of convergent evolution. The work illustrates a fundamental difference in the substrate-binding mode between Mn^2+^ dependent isatin hydrolase like enzymes in comparison with the vast number of Zn^2+^ dependent enzymes.

## Introduction

The catalytic activity of metallohydrolase enzymes strongly depends on the identity of the metal and on the nature of its binding site. Zn^2+^ bound hydrolytic enzymes, in particular metallo-β-lactamases^1^, probably constitute the most extensively studied cases for such a group. Depending on the enzymatic class, hydrolytic enzymes with both a mononuclear or dinuclear Zn^2+^ sites have been identified as widely distributed throughout all kingdoms of life^2^. The catalytic mechanism for the mononuclear and dinuclear Zn^2+^ sites have been debated for years^3–5^, and only recently the collection of accumulated knowledge allowed the proposition of a plausible mechanism^6–9^. Despite Zn^2+^ being the most widely distributed hydrolytic cation, it has been shown that Zn^2+^ also possesses an inhibitory potential for certain metallohydrolases as, for example, in carboxypeptidase A^10^.

The specialized metallo-enzyme amidohydrolase superfamily (AHS) is able to carry out a diverse range of chemical reactions including the degradation of metabolic precursors like pyrimidine (dihydropyrimidinases) and hydantoin, (hydantoinases)^11^. The AHS members are often categorised as both Zn^2+^ and Mn^2+^ dependent hydrolases. However, amidohydrolases involved with purines degradation in both bacteria and plants are mainly Mn^2+^ dependent allantoinases^12,13^, even though Zn^2+^ dependent allantoinase activity has been reported^14^.

The compound isatin is a metabolic precursors and was originally identified as an intermediate product of the indole-3-acetic acid (IAA) degradation pathway in *Bradyrhizobium diazoefficiens*^15^. Hydrolytic activity of isatin hydrolase (IH) was first demonstrated in various symbiotic rhizobial species^16^. Recent bioinformatics studies indicates that IH are found widespread in bacteria including pathogens specific to the human gut and plants, e.g., *B. enterica* and *R. solanacearum*^17^. Isatin hydrolase A from *Labrenzia aggregata* (*La*IHA) and *R. solanacearum (Rs*IHA) share 59% sequence identity while the two putative orthologues *La*IHA and *La*IHB in *L. aggregata* share 51% sequence identity. Both *La*IHA and *La*IHB contain the central metal binding motif HxG[T/A]HxDxPxH in each protomer of the catalytically active dimer. Structural characterization of *La*IHB revealed a novel fold, which later was also attributed to other amidohydrolases such as bacterial kynurenine formamidase B (KynB) (EC 3.5.1.9)^17^. The fold is generally described as an α/β hydrolase with a central scaffold resembling the swivelling β/α/β fold^18^, while the majority of AHS members contains a monomeric (β/α)_8_-TIM like-barrel structural fold^19^. A notable difference between *La*IHB and kynurenine formamidase from *Bacillus anthracis* (*Ba*KynB) is the presence of a mononuclear manganese-binding site in *La*IHB, while the structure of KynB accommodates a bi-nuclear zinc site^20^. *La*IHB shows a strict specificity for Mn^2+^ over Zn^2+^, as reported by kinetic measurements^17^, and rationalised by computer simulations^21^.

Here we present two crystal structures of *La*IHA, one bound to the hydrolytic product isatinate, and one bound to benzyl benzoate, as well as the apo structure of homologous *Rs*IHA. Together with results from enzyme kinetic analysis, the resolved structures are used to describe the molecular basis for the Mn^2+^-dependent mechanism of hydrolysis, showing that IHA is a true orthologue of IHB. For the first time, the mechanism of a Mn^2+^ dependent hydrolysis reaction is characterised in detail by computer simulations using multiscale quantum mechanics/molecular mechanics (QM/MM)^22^ and *ab initio* molecular dynamics (MD) simulations^23^. The simulations were crucial to unravelling the essential catalytic role of the conserved residues His79 and His207 for the formation of the product. Based on the identification of mechanistically important residues presented in this work, including the Mn^2+^ binding residues in the *La*IHA active site, we further present evidence that the Mn^2+^ dependent allantoate amidohydrolase (AAH) (E.C. 3.5.3.9) displays a striking similarity in the active site geometry despite the differences in the overall protein fold. As this likely represents a case of convergent evolution, we propose that the generalised catalytic mechanism described here for the IHs may also apply to the AAH family and other similar Mn^2+^ dependent hydrolases.

## Materials and Methods

### Cloning, expression and purification of *La*IHA and *Rs*IHA

Chemicals were purchased from Sigma-Aldrich (Norway) unless stated otherwise. The open reading frame encoding *La*IHA (UniProtKB: A0P0F0) was amplified by PCR from a boiled colony of *L. aggregata* IAM12614. DNA fragments were isolated and digested with *Bam*HI and *Eco*RI and cloned into the T7-RNA polymerase dependent *E. coli* expression plasmid pT7H6^24^. The 6xHis containing *La*IHA was expressed in *E. coli* BL21 AI cells (Invitrogen) in 2xTYE-medium for 4 hours at 37 °C. Cells were harvested by centrifugation, re-suspended, and lysed by sonication in a buffer containing 50 mM Tris-HCl pH 8.0 and 0.5 M NaCl supplemented with a protease inhibitor tablet (Roche cOmplete, EDTA-free). Insoluble material was removed by centrifugation (4000 *g*, 10 min.). The soluble protein extract was batch-adsorbed onto 25 mL Ni-NTA agarose resin (Qiagen) per litre of original culture and loaded into liquid chromatography columns. The protein loaded Ni-NTA columns were washed with >20 column volumes (CV) of equilibration buffer (50 mM Tris-HCl pH 8.0, 500 mM NaCl, 50 mM imidazole). The protein was eluted from the resin with elution buffer (50 mM Tris-HCl pH 8.0, 500 mM NaCl, 10 mM Na_2_EDTA). Fractions containing the recombinant proteins were pooled, and the buffer changed into a low salt buffer (10 mM Tris-HCl pH 8.0, 100 mM NaCl, and 1 mM Na_2_EDTA) on a Sephadex G-25 column (GE Healthcare). Before crystallisation and kinetic experiments, *La*IHA was further treated with 10 mM EDTA and dialysed (500 fold dilution) into a stability buffer (5 mM bis-tris pH 7.0, 100 mM NaCl and 1 mM DTT). The protein solutions were centrifuged at 180,000 *g* for 10 minutes at 4 °C, and the supernatant could hereafter be stored in a stable condition at 4 °C for at least 12 months.

The *Rs*IHA (UniProtKB: Q8XYC3) was ordered from Genscript and inserted into the expression vector pET-M11. The six-histidine containing construct was expressed in *E. coli* BL21 gold cells, in LB-medium (containing 50 µg/mL kanamycin) overnight at 18 °C. Cells were harvested by centrifugation, re-suspended, and lysed by a bead-beater (Biospec) in a lysis buffer (50 mM Tris-HCl pH 8.0 and 0.1 M NaCl), protease inhibitor tablet (Roche cOmplete, EDTA-free). Insoluble material was removed by centrifugation, and the lysate was supplemented with 20 mM imidazole before loaded onto a pre-equilibrated HisTrap HP (GE). The column was washed with 10 CV with equilibration buffer (50 mM Tris-HCl pH 8.0, 0.1 M NaCl, and 20 mM imidazole) and eluted with elution buffer (50 mM Tris-HCl pH 8.0, 0.1 M NaCl, and 300 mM imidazole). The pooled fractions were supplemented with a molar 1:50 ratio of tobacco etch virus (TEV) protease and dialysed overnight against dialysis buffer (50 mM Tris-HCl pH 8.0, 100 mM NaCl). The *Rs*IHA was again loaded to the HisTrap HP column and the flow-through was collected. *Rs*IHA was concentrated to 20 mg/mL and loaded onto a Superdex 200 10/300 GL size exclusion column (GE) pre-equilibrated in SEC buffer (50 mM Tris-HCl pH 8.0, 100 mM NaCl). Pure *Rs*IHA were pooled, concentrated, and stored at −80 °C. *Rs*IHA was treated like *La*IHA before crystallisation and kinetic experiments.

### Crystallization of *La*IHA and *Rs*IHA ligand complexes

The pre-treatment of *La*IHA and *Rs*IHA with an excess of EDTA and dialysis was mainly carried out to remove any potential trace of metals, leaving an estimated EDTA concentration at 0.02 mM. Prior to crystallization 1 mM MnCl_2_ was added. *La*IHA and *Rs*IHA were crystallized at 22 °C using a protein concentration of 15 mg/mL in 5 mM Bis-Tris pH 7.0, 100 mM NaCl, and 1.0 mM MnCl_2_. *La*IHA was crystallised by vapour diffusion using sitting drops 1 + 1 μL (*La*IHA: Reservoir) over 100 μL reservoir solution. The best diffracting crystals were obtained in 20% glycerol and 20% PEG 1500. The *La*IHA:isatinate structure was obtained by soaking the crystals for approximately 1 min with 500 µM isatin before flash-freezing the crystals in liquid nitrogen (LN_2_). This condition also served as cryo-protection. *Rs*IHA was also crystallised with vapour diffusion using hanging drops, 1 + 1 μL (*Rs*IHA: Reservoir) over 400 μL reservoir solution sealed with immersion oil (56822, Sigma). The best diffracting crystals of *Rs*IHA grew in 1 M succinate, 100 mM HEPES pH 6.5. Cryo-protection was achieved by adding 1 μL of 60% w/v PEG400 suspended in reservoir solution to the drop. The crystals were mounted and flash-cooled immediately after. Crystals of *La*IHA:benzyl benzoate was obtained in 28% PEG 1500 and 1 mM MnCl_2_ with a hanging drop setup using 250 μL reservoir and 1 + 1 μL drops. With a protein concentration of 20 mg/mL used. The hanging drop wells were sealed with immersion oil. Crystals appeared within one week. Cryo-protection was obtained by adding glycerol to the reservoir solution to a final concentration of ~12%, which after 12 hours would allow additional vapor diffusion to take place before mounting and flash-cooling in LN_2_^25^.

### Data collection and structure determination

All datasets were collected at 100 K. For *La*IHA:Benzyl benzoate a dataset extending to 1.50 Å resolution was collected at a wavelength of 1.0004 Å. The space group was determined to be *P*1, and a collection strategy was calculated by iMOSFLM^26^. The data were processed with XDS^27^. A test set of 5% was used for *R*_*free*_ calculation^28^. Phasing was performed by molecular replacement (MR) using *La*IHB (PDB ID: 4J0N) as a search model for Phaser^29^. Initial model building and refinement were performed in Phenix^30^. The final model was built using Coot^31^ with small ligand models and restraints produced using eLBOW^30^. For *La*IHA:Isatinate, a diffraction dataset was collected at beamline P13 at PETRA III (Hamburg, Germany)^32^. The space group was determined and would only allow a *P*1 lattice. A complete dataset was collected at 1.79 Å resolution with and oscillation range of Ω = 0.01° and processed with XDS. The structure was solved with MR using a *La*IHA: Benzyl benzoate monomer as the search model and performed with Phaser in Phenix. Diffraction data on *Rs*IHA was collected at beamline ID30B at ESRF (Grenoble, France). The space group was determined and allowed *P* 3_2_ 2 1, a complete dataset with at Ω = 0.05° over 126° was collected. The data extended to 2.65 Å and was processed with XDS. The structure was solved with MR using *La*IHA:Benzyl benzoate monomer as the search model in Phenix. Model building and refinement was performed using coot^33^ and phenix.refine within the phenix package^30^. NCS restraints were applied throughout the refinement, however released in the final round (Number of molecules in the asymmetric of each crystals structure have been added in the Supplementary Table S1)^34^.

All structure validations were performed using MolProbity^30^. Structure analysis and figure preparation were done using PyMOL Molecular Graphics System, Version 1.5.0.3, Schrödinger, LLC. The finalised model and structure factors were deposited to Protein Data Bank (PDB) and given the PDB accession codes (*La*IHA:Benzyl benzoate PDB ID 5NNA; *Rs*IHA PDB ID 5NMP; *La*IHA:isatinate PDB ID 5NNB).

### Enzymatic assay

The measurement of the activity, and its metal dependence, were performed as described by Bjerregaard-Andersen *et al*.^17^. The method is based on an increased absorption of the product isatinate at 368 nm. This can be followed using a standard spectrophotometer. Measurements were performed using a JASCO V-630 spectrophotometer with a cuvette light path of 1.0 cm. An extinction coefficient of 4.5 × 10^3^ cm^−1^mol^−1^L was used for isatinate. The data were fitted with the Michaelis-Menten equation assuming one binding site using the software GraphPad Prism 6.

### Sequence analysis

Structural homologues of *La*IHA and *E. coli* allantoate amidohydrolase (*Ec*AAH) were identified using the DaliLite v. 3^35^, followed by inspection in PyMoL. The sequences of the identified homologues were then imported in Jalview^36^ using the fetch from PDB option and combined with sequences of *La*IHA, *Rs*IHA, and *Rhodococcus rhodochrous* HpoH (*Rr*HpoH). Sequences with the following UniProt accession codes (in parentheses) were used: *La*IHA (A0P0F0), *La*IHB (A0NLY7), *Rs*IHA (Q8XYC3), *Ec*KynB (B4E9I9), *Pa*KynB (Q9I234), *Ba*KynB (Q81PP9), *Bs*AHD (P84132), *Mj*AHD (Q58193), *Rr*HpoH (B5MAD9). For AAH alignment, the following accession codes were used: *Ec*AAH (P77425), *At*AAH (Q8VXY9), *Bv*HYD (A4JQA0), *Bm*HYD (A0A0H3KRF1), *Bc*HYD (B4EHA1), *Ge*RAC (Q53389), *Sk*SYN (Q96W94). Sequence alignments were performed using the Clustal algorithm with default settings in Jalview. Phylogenetic trees were calculated in Jalview as average distance using identity percentage and exported to TreeDyn 198.3^37^ for visualisation. The sequences were included in alignment as supplied by the PDB.

### Molecular modelling

The system consisting of *La*IHA, isatin, 47331 water molecules, and 19 Na^+^ ions was placed in a 105 × 126 × 118 Å^3^ simulation box. The initial geometry of the protein was taken from the X-ray structure of *La*IHA:isatinate resolved by us and described in this text. Isatin was introduced in the system by molecular replacement, fitting the aromatic moiety of isatinate. The Mn^2+^ ion, isatin, the catalytic water, the side chains of the ligating amino acids (His69, His73, Asp75, Gln219), and of the neighbouring His79, His207, Asp193 residues were treated at a quantum-mechanical level (QM) using density functional theory with the Becke and Lee-Yang-Parr (BLYP) approximations for the exchange-correlation functional^38,39^. The Kohn-Sham orbitals for the valence electrons were expanded over a DZVP Gaussian basis set^40^ and an auxiliary plane-wave basis set with a cut-off of 240 Ry. The core electrons were integrated out using Goedecker-Teter-Hutter pseudopotentials^41^. The remainder of the system was described at the molecular-mechanics (MM) level using the Amberff14SB force field^42^.

In a first step, the system was equilibrated by full classical molecular dynamics (MD) simulations in the NpT ensemble (T = 300 K, p = 1 atm), constraining the QM region to its initial positions, and using a time-step of 1.5 fs. Thereafter, QM/MM Born-Oppenheimer MD simulations were performed at 300 K within the NVT ensemble using the Nosé–Hoover thermostat^43–45^ and a time-step of 0.25 fs. The simulations were run using the CP2K package (https://www.cp2k.org/)^46–48^. The mechanism of hydrolysis was sampled by coupling QM/MM MD to metadynamics simulations^49,50^ using collective variables defined as differences between coordination numbers (Visualized in Fig. S1, in the supporting information):

$$\begin{array}{rcl}{\rm{CV1}} & = & {\rm{CN}}\,({{\rm{C}}}_{{\rm{isatin}}}:{{\rm{N}}}_{{\rm{isatin}}})-{\rm{CN}}\,({{\rm{C}}}_{{\rm{istatin}}}:{{\rm{O}}}_{{\rm{water}}});\\ {\rm{CV2}} & = & {\rm{CN}}\,({{\rm{N}}}_{{\rm{His83}}}:{\rm{H}})-{\rm{CN}}\,({{\rm{O}}}_{{\rm{water}}}:{\rm{H}});\\ {\rm{CV3}} & = & {\rm{CN}}\,({{\rm{N}}}_{{\rm{istatin}}}:{\rm{H}})-{\rm{CN}}\,({{\rm{N}}}_{{\rm{His212}}}:{\rm{H}}),\end{array}$$

where CN is the coordination number between two atoms. Each CN is defined as:

1 $$CN(i:j)=\frac{1-{({d}_{ij}/{d}_{0})}^{p}}{1-{({d}_{ij}/{d}_{0})}^{p+q}}$$

where ${d}_{ij}\,\,$ is the distance between atoms $i$, $j$, and ${d}_{0}$, p, and q are free parameters. In our simulations, ${d}_{0}$ was set to 1.6 Å for CV1, and 1.1 Å for CV2 and CV3. Values of $p$ = 12 and $q$ = 14 were used for all the three CVs. The d0, p, q values where chosen so that the profile of the sigmoidal function (equation 1) did not change significantly during a standard thermal oscillation of the bond, while it signalled the formation or breaking of a chemical bond. Gaussian hills with a height of 2 kcal/mol were spanned every 100 step (i.e., every 25 fs). A Gaussian spread of 0.15 was used for all three collective variables. The free energy profile for the reaction was obtained as the sum of the Gaussians added during the whole metadynamics simulation. The three CVs used in this study were chosen to take into account both the nucleophilic addition/elimination at the carbonyl group and the proton rearrangements necessary to yield the product. Runs using any combination of only two of these CVs did not produce any reactive profile, indicating that all the three CVs are necessary for the determination of the reaction coordinate.

With this setup, the system reacted after 1.1 ps of metadynamics simulations, the run was continued until a total time of 1.85 ps to reach convergence in the free energy profile. We assumed convergence of the profile after observation of a second re-crossing of the barrier (Supplementary Fig. S2).

## Results

### IHA contains a mononuclear manganese binding site involving an additionally conserved glutamine

**Figure 1:**
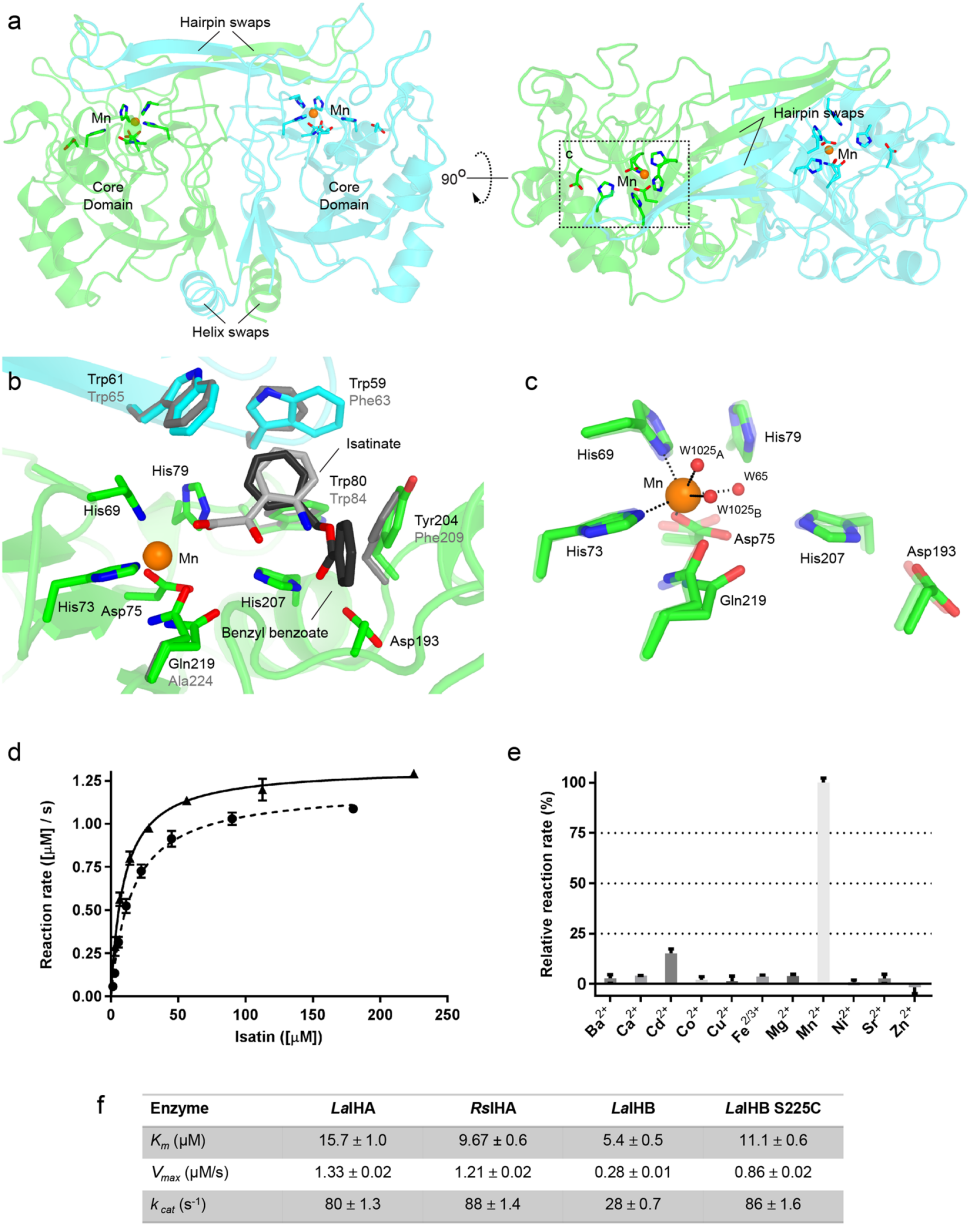
(**a**) Overall structure of the *La*IHA dimer found in the asymmetric unit, the protomers are colored green and cyan and shown in two different orientations rotated by 90 degrees. Manganese and highlighted residues a location of active sites in the dimer. (**b**) Isatinate and benzyl benzoate in the substrate binding pocket of *La*IHA. Isatinate is coordinating bidentate directly to the manganese. Parts of the pocket, residues Trp61 and Trp59, are contributed by the opposing monomer via the hairpin swap. Key binding pocket residues of the *La*IHB binding pocket (grey) are functionally conserved. (**c**) Catalytically important residue in the active site of *La*IHA: benzyl benzoate. The corresponding site of *La*IHA: benzyl benzoate is superposed (transparent). The manganese is found in octahedral coordination similarly to that described in^17^. Gln219 resides in a double conformation and only partially coordinates to the manganese. Also, W1025 is found in a double conformation (denoted A and B in Fig. 1). Note that Asp75 is coordinating bidentate in *La*IHA:isatinate while monodentate in *La*IHA: benzyl benzoate. (**d**) The isatin hydrolysis by *La*IHA (circles) and *Rs*IHA (triangles) follows Michaelis-Menten kinetics, and the parameters are collected in f). (**e**) Both *La*IHA and *Rs*IHA display strong manganese dependency. (**f**) Kinetic parameters of *La*IHA and *Rs*IHA collected with previous values from *La*IHB and activated mutant *La*IHB S225C. All measurements in (**d**) and (**e**) were performed in triplets.

The structures of *La*IHA:benzyl benzoate and *La*IHA:isatinate were determined at 1.50 Å and 1.79 Å resolution, respectively, while the one of *Rs*IHA was determined at 2.65 Å resolution. Data collection and model refinement statistics are collected as described in Supplementary Table S1. Both *La*IHA structures are highly similar to the one of *La*IHB with a root mean square deviation (rmsd) value of ~0.7 Å computed on all main chain atoms. Both *La*IHB and *La*IHA are dimers with two exchanged regions – a small N-terminal α-helical exchange and a larger β-hairpin exchange (Fig. 1a). A hallmark of this fold is the contribution of two conserved residues of one monomer in the formation of the substrate-binding pocket located in the other monomer. For the IH’s these two residues are two tryptophans (Fig. 1b).

**Figure 2:**
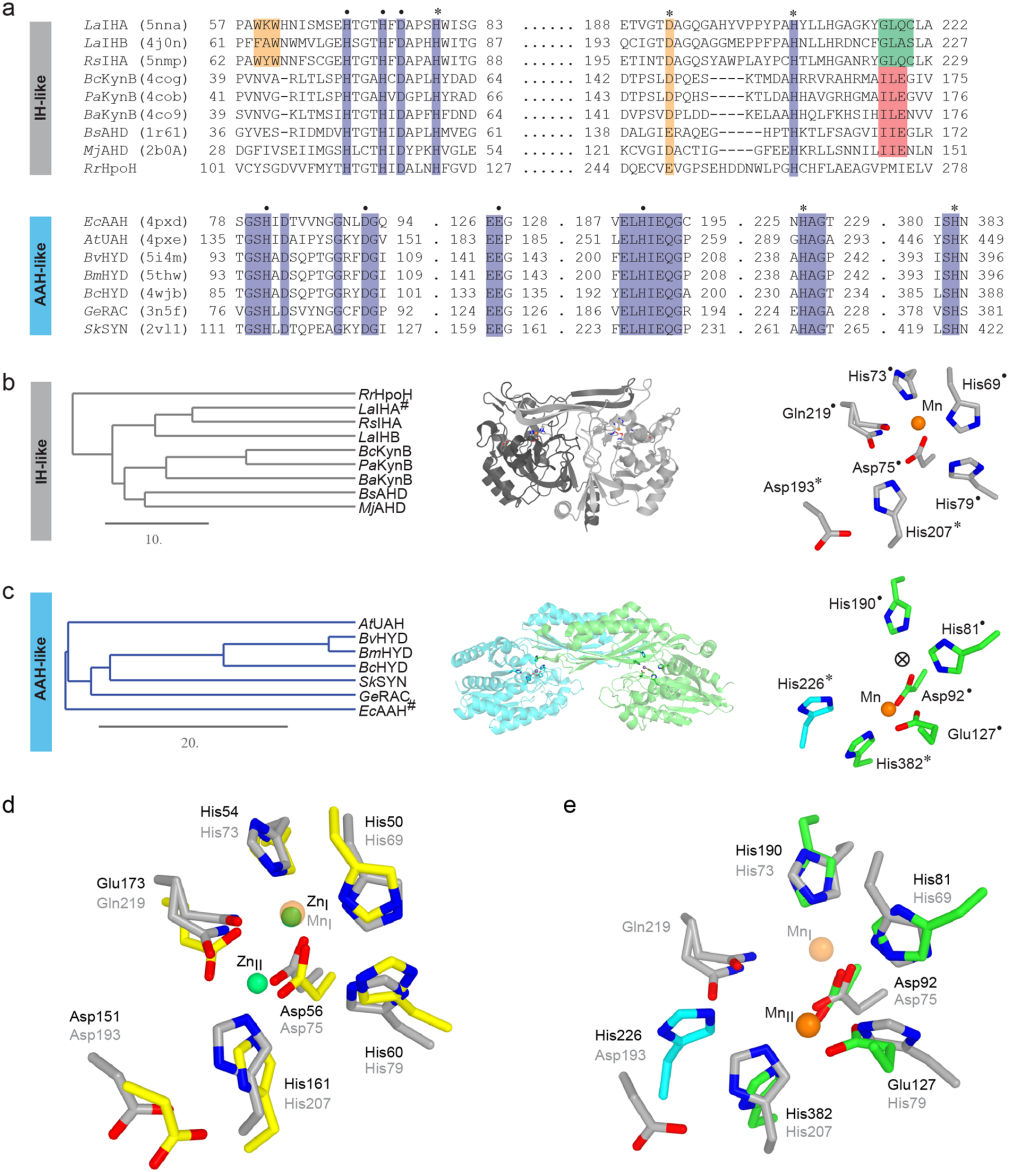
(**a**) Alignment of selected sequences of IHA and AAH homologues with known structures. Conserved residues are highlighted in blue. Residues involved in metal coordination are indicated by a dot, residues presumed to be involved in proton transfer are indicated with an asterisk. All are completely conserved, apart from the *La*IHA Asp193 (yellow) which is functionally conserved. The orthologue is defining aromatic binding pocket motif for isatin, WXW/FXW, is highlighted in orange. The key GLQC/GLAS motif of IHA and IHB, indicating isatin hydrolase activity, is highlighted in green, with a similarly conserved motif in KynB homologues highlighted in red. (**b**) Phylogram of IHA homologues from A indicates IHA-like and KynB-like sequences, *Rr*HpoH proposed to carry a novel cyclase activity seems to belong to neither of these. An example of the fold (dark and light grey) and active site position is shown. (**c**) Phylogram of AAH homologues. Note the large diversity between *Ec*AHH (prokaryote) and *At*AAH (eukaryote). An example of the fold (green and cyan) and active site position is shown, His226 (*At*AAH) is delivered from opposing monomer through domain swap of *At*AAH. (**d**) Active site residues of *Ba*KynB (Yellow, black numbers) superposed with *La*IHA (Grey, grey numbers), the mononuclear Mn^2+^ in *La*IHA is labelled (Mn_I_) and the binuclear Zn^2+^ sites in *Ba*KynB are labelled Zn_I_ and Zn_II._ (**e**) Active site residues of *Ec*AAH (green and cyan, black numbers) superposed manually with *La*IHA (grey, grey numbers), the mononuclear Mn^2+^ from *Ec*AAH labelled Mn_II_.

The highly conserved Mn^2+^ binding site is located at the bottom of the substrate-binding pocket. The Mn^2+^ is found in an octahedral complex coordinated by His69, His73, Asp75, and two water molecules (Fig. 1c). Asp75 forms a bidentate contact in *La*IHA:Benzyl benzoate and a mono-dentate electrostatic contact in the *La*IHA:isatinate structures. The mono or bi-dentate coordination results in two or one water molecules as additional ligands, respectively, preserving the octahedral geometry. The side chain carbonyl of Gln219 in *La*IHA (Ala224 in *La*IHB) is found in alternative conformations, one of which coordinates Mn^2+^. The non-binding conformation allows additional water to approach to Mn^2+^ (W1025_A/B_, Fig. 1c). Sequence analysis reveals that Gln219 is part of a motif, conserved as either GLAS (e.g. in *La*IHB) or GLQC (e.g. in *La*IHA) (Figs 1 and 2a). The short residues motifs AS and QC are conserved as sequence pairs, which could indicate a functional dependency. Interestingly, the residues S or C are found to be the key residue involved in regulating the proton flow through the water channel. A mutation of the serine residue to a cysteine to form a GLAC motif caused a gain in activity in *La*IHB^17^. The GLQC/GLAS sequence, combined with the conserved manganese binding site appears to be a signature motif for isatin hydrolase activity. In KynB, the comparable position holds a conserved I(L/I)E motif, which is also found in the two uncharacterized structures of amidohydrolases (AHD) from *Bacillus stearothermophilus* (*Bs*AHD) and *Methanococcus jannaschii* (*Mj*AHD) (Fig. 2a, Full sequence alignment shown in Supplementary Fig. S3). The motif is absent in *Rr*HpoH, which has confirmed cyclase activity. Whether the I(L/I)E motif and perhaps, in particular, the glutamate is indicative of activity on linear amides or a binuclear metal site remains uncertain.

Activity measurements confirm that both *La*IHA and *Rs*IHA have isatin hydrolase activity (Fig. 1d,f). The determination of the catalytic parameters assuming Michaelis-Menten kinetics yields *K*_*m*_ values for *La*IHA and *Rs*IHA of 16 μM and 10 μM, respectively. Structural superposition revealed identical metal binding sites in the two homologues. Thus, a metal dependence analysis was only performed on *La*IHA. *La*IHA shows the highest activity in the presence of Mn^2+^ confirming that this enzyme is Mn^2+^ dependent. However, the relative activity of 15.1 ± 2.2% is also observed in the presence of Cd^2+^. Notably, *La*IHA could not be activated in the presence of both Zn^2+^ and Cu^2+^ (Fig. 1e).

### QM/MM calculations reveal the importance of an Asp-His dyad in the proton abstraction

**Figure 3:**
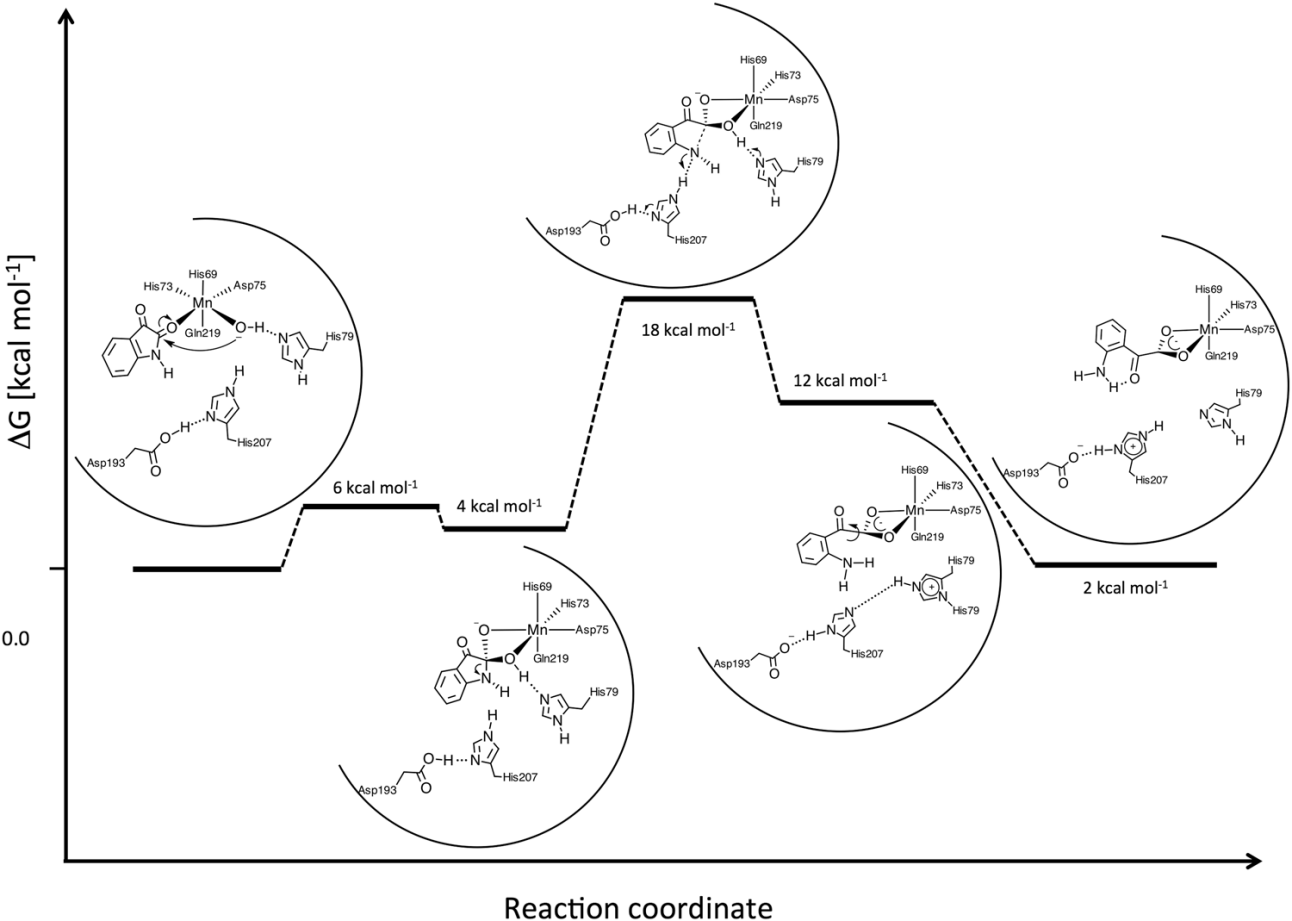
Reaction mechanism describing the hydrolysis of isatin. The reaction is visualised from the initial nucleophilic attack of OH^−^ to the carbonyl of isatin until the final substrate isatinate is formed. The final step involves isatinate isomerization, promoted by the strong intra-molecular H-bond between its amino and α-carbonyl groups, as well as the reformation of the hydrogen bond between Asp193 and His207. Free energy differences were estimated from metadynamics simulations.

In *La*IHA, both the carbonyl group of the substrate and the catalytic water coordinate to the Mn^2+^ ion. Like in other hydrolytic enzymes, the nucleophilic water is activated by a pre-organized base, which can efficiently extract a proton to form hydroxide (OH^−^). In *La*IHA, His207 acts as such base. The positively charged protonated His207-H^+^ is stabilised by the presence of a hydrogen-bonded partner, i.e., Asp193. The water-His207-Asp193 H-bond network closely resembles the Ser-His-Asp triad common to serine proteases. His79, also H-bonded to the catalytic water, completes a complex His79-water-His207-Asp193 proton-shuttle system (Fig. 2b,d). Water deprotonation in *La*IHA is extremely efficient and occurs spontaneously during the QM/MM simulations at room temperature^21^. Activation of the catalytic water is favoured by its isolation from the water channel that connects the substrate cavity to the exterior. Confinement of the catalytic water has been reported as an important structural requirement in other hydrolytic (metallo)-enzymes^51–55^. Figure 3 depicts the molecular details of the hydrolytic reaction. The nucleophilic attack of OH^−^ to the carbonyl of isatin leads to the formation of a tetrahedral intermediate. Our model predicts an activation barrier of a few (i.e., less than 6) kcal mol^−1^. Thus, as in other metallohydrolases (i.e., zinc-β-lactamases), this step is not rate-limiting for the reaction. The tetrahedral intermediate is only metastable and it can easily recombine into the reactant state. In fact, the following separation of the scissile C-N bond constitutes the rate-limiting step of the reaction with a barrier height of ~12 kcal mol^−1^.

Our data report that C-N separation occurs through a dissociative pathway. The transition state is stabilised by formation of two hydrogen-bonds (H-bonds), the first between the nucleophilic OH^−^ and the Nδ atom of His79, and the second between the isatin N atom and the Nε-H moiety of His207 (Fig. 3, Supplementary movie). The final product formation of isatinate is accompanied by synchronous proton exchange in both of these two H-bonds. Our simulations indicate that the proton transfers occur after the transition state has been reached and the C-N bond is broken. Kinetic measurements in a deuterated environment did not reveal any observable kinetic isotope effect on the reaction, providing an additional indication that proton transfers are not involved in the rate-limiting step of the enzymatic process (data not shown).

The preference of a dissociative pathway over an associative or concerted mechanism may be a consequence of the fact that the N-atom in isatin acts as a poor base. In general, the π conjugation between the lone pair of N and the aromatic ring present in aryl-amines decreases the *pKa* value by few pH units. As a result, we observe that formation of a stable H-bond with His207, and consequently the proton transfer, occurs only upon a major elongation of the isatin C-N bond, which is associated to increased localization of the electron density at the N atom. Additional stabilization of the moiety into the product is obtained by the deprotonation of the nucleophile OH^−^ group by His79 to produce a carboxylate group chelating the Mn^2+^ as observed in the crystal structure.

The isatinate conformer produced by hydrolysis is not the most stable one. In fact, the α-carbonyl can isomerise via a bicycle motion, similar to that described in excited state isomerisation of retinal in rhodopsin^56^. This isomerised product found in our simulations is in agreement with the binding geometry of the product, isatinate, described in the crystal structure (Fig. 1b). In particular, the final binding state of isatinate shows bidentate coordination to the manganese ion, and similar stacking interactions with the indole rings of Trp59, Trp61, Trp80 and Tyr204 (Fig. 1b).

The strong exothermic isomerisation step (−10 kcal mol^−1^) yields a structure of isatinate with a strong intra-molecular H-bond between the amino and the α-carbonyl groups. This possible intermediate most likely constitutes the driving force of the reaction and traps the isatinate as a ligand to Mn^2+^ in its product state before it is replaced by water and released from the binding pocket (Fig. 3).

### Isatin hydrolase and kynurenine formamidase active-site conservation indicates a similar mechanism

The recently determined crystal structure of kynurenine formamidase from *Bacillus anthracis* (*Ba*KynB) contains a binuclear Zn^2+^ site and thus is part of the EC 3.5 class that has a similar overall protein fold when compared with *La*IHA (sequence identity 24%). Figure 2d shows the superposition of the metal binding sites for *La*IHA:isatinate and *Ba*KynB (PDB ID: 4COG). The structural architecture of the metal binding sites is highly conserved between both enzymes (Fig. 2d). The most striking difference is the position of Gln219 in *La*IHA, which is occupied by glutamate (Glu172) in *Ba*KynB. This residue is conserved for all verified KynB enzymes. In *Ba*KynB, the His60 (equivalent to His79 in *La*IHA) is proposed to be necessary for the hydrolysis reaction to take place, by accepting a proton from the bi-coordinated activated water. In the mechanism for the isatin hydrolysis presented here, both His79 and His207 are involved in the catalytic mechanism (Supplementary Movie M1). In *Ba*KynB, the Zn^2+^ in position (I) Zn_I_^2+^ is octahedrally coordinated and bound in an equivalent position as Mn^2+^ in *La*IHA. In a homologous structure of KynB from *Burkholderia cenopacia* (*Bc*KynB), this position is occupied by Cd^2+^. The Zn^2+^ in position (II), Zn_II_^2+^ is also found in an octahedral conformation.

### Convergent evolution of the active-site geometry in *La*IHA and *Ec*AAH

Amidohydrolases with known manganese dependency where identified through literature searches and examined manually for structural similarity. This approach revealed two candidates that, upon close inspection, shared metal binding sites residues and residues identified as catalytically important. The allantoate amidohydrolases (AAHs) are part of the purine degradation pathway and hydrolyse the allantoate to ureidoglycolate, carbon dioxide, and ammonium in the process, the allantoin pathway is found in both plants^13^ and bacteria^57^ (Fig. 2a,c).

The identified structures were manually superposed with the active site of representatives from the AAH’s, (Fig. 2e). The structure for *E. coli* AAH (*Ec*AAH, PDB ID: 4PXD) was used as input for the DALI server^35^. The found hits were visually inspected for structural homology and included in alignment to confirm residue conservation (Fig. 2a).

## Discussion

The Baltic sea bacteria *Labrenzia aggregata* genome includes two homologous open reading frames for isatin hydrolases A and B that share 51% sequence identity (SI). The more distant homolog from *Ralstonia solanacearum* (*Rs*IHA) shares 59% SI with *La*IHA and 51% SI with *La*IHB. Unlike the whole sequence, the active site and substrate binding pocket are highly conserved among these proteins. Even though the key substrate binding pocket residues originally identified in the structure of *La*IHB - Phe63, Trp65 and Trp84 and Phe209^17^ do not share equivalent positions in the sequences of *La*IHA or *Rs*IHA, their aromatic side chains do adopt an identical structural conformation in the respective binding pockets, thus indicating functional conservation (Fig. 1b). Compared to *La*IHB, the Mn^2+^ binding sites in both *Rs*IHA and *La*IHA include Gln219, also responsible for increased metal specificity^21^. The crystal structure of *Ba*KynB from *Bacillus anthracis* (PDB ID: 4COG) presents a glutamate (Glu173, Fig. 2d) adopting an equivalent conformation as that of Gln219 in *La*IHA. In conjunction with the conserved His161 and Asp56 (*Ba*KynB numbering), it forms a secondary Zn^2+^ site (Zn_II_) binding site in the presented binuclear metal binding (Fig. 2d). The remaining metal-coordinating residues are identical to both *Rs*IHA and *La*IHA. It cannot be completely ruled out that, in certain conditions, Gln219 would allow the formation of a secondary metal-binding site in *La*IHA. This is however unlikely, due to the different Lewis-acid/base properties of an amide group, compared to those of a carboxylate. The structure of *La*IHB, which features an alanine residue in the position of Gln219, does not have this option and thus is expected to be strictly mononuclear, with the highest hydrolytic activity measured in the presence of Mn^2+^, as also observed for *La*IHA presented in this manuscript.

In the case of the amidohydrolase diaminopimelate desuccinylase (dapE), it was found that the compound L-captopril only binds to the Zn^2+^ bound form of the enzyme and not to the physiologically relevant Mn^2+^ bound form, thus stressing the importance of identifying the relevant metal^58^.

Different metallo-β-lactamases can bind and be catalytically active in the presence of both one and two Zn^2+^ metals in the binding site. In both cases, the reaction mechanisms were characterized by computational modelling in the past (1 Zinc: B1 BcII from *Bacillus cereus* and B2 CphA from *Aeromonas hydrophila*, 2 Zinc: B1 CcrA from *Bacteroides fragilis*)^59–61^, the mono and binuclear reaction scheme for Zn^2+^ binding beta-lactamases has recently been revised in Lisa MN *et al*.^6^. The mechanism of hydrolysis in *La*IHA shares similarities with those found in single-Zn^2+^ enzymes. In particular, the reaction proceeds through a two-step mechanism, where the nucleophilic attack on the amide carbonyl of isatin is followed by the breaking of the C-N bond. On the contrary, in CcrA from *B. fragilis*, the reaction follows a concerted mechanism where the nucleophilic attack of the hydroxyl takes place simultaneously with the opening of the β-lactam ring.

Common to all these enzymes is that the rate-limiting second step of the reaction is constituted by the dissociation of the C-N bond, and that the pathway to the product is facilitated by proton transfer on the N atom that usually occurs at the transition state or immediately after. The proton-shuttling pathways are nonetheless different in the Zn-bound enzymes and IH. The lone pair of the amidic nitrogen of isatin is π-conjugated with both the amidic carbonyl and the aromatic phenyl ring present in the molecular structure. As a consequence of the resulting electronic delocalisation, its basicity remains low even after the initial attack of the OH^−^ and the subsequent formation of the tetrahedral intermediate. In fact, the evolution from the TS to the product requires both the pre-organised His207-Asp193 dyad shuttling one proton on the N atom and the stabilisation of C-N heterolytic breakage by a secondary proton transfer from the carboxylic group to His79. As a consequence, again differently from metallo-β-lactamases, the product formation in *La*IHA leaves the protein in a higher protonation state than the initial complex. The deprotonation of the active site after the release of the product likely occurs through the water channel originally described for *La*IHB^17^. Our *in silico* studies yield further support to the necessity for proton removal, in order to recover the catalyst, and allow further enzymatic cycles.

As the calculations produced a slightly endothermic profile (+2 kcal), the excess energy released by deprotonation of the protein may also constitute the driving force for the enzymatic process. Though, we stress that due to high computational costs, the free energy profile was obtained with a resolution that is of the same order as the reaction free energy balance.

The sequence alignment and structural superposition confirm that not only the metal coordinating residues (Fig. 2a–c, dots), but also the residues involved in the proton transfer (Fig. 2a–c, asterisk) are highly conserved. Only the position corresponding to *La*IHA residue Asp193 (Fig. 2a) alternates between aspartate and glutamate, thus showing anyway functional conservation. The conservation suggests functional importance and, supported by the structural superposition and mechanistic elucidation, points to a common catalytic role. This is particularly interesting in the light of the sequence variation within the IH-like and AAH-like folds as illustrated in Fig. 2b,c,e. Here, the phylogenetic tree of proteins belonging to the respective folds indicates a large sequence variation, perhaps best exemplified by the significant divergence of *Ec*AAH compared to *Arabidopsis thaliana* AAH (*At*AAH), i.e., prokaryotic and eukaryotic. However, both the overall fold, displaying domain-swapped dimers, and the active site geometry are highly conserved (Fig. 2c–e).

It remains to be established whether the catalytic mechanism exemplified in *La*IHA by our structural and *in silico* studies is specific to the IH-like enzymes, or could represent a broader case of convergent evolution across a range of AHS metallo-hydrolases. In the case of the AAH homologues, the Mn^2+^ binding site is structurally completely conserved, including the binding residues in the Mn_I_ site (His69, His73 and Asp75 for *La*IHA), and the proton transfers triad (His79, His203 and Asp193) in *La*IHA compared to Glu127, His382 and His226 in *Ec*AAH, with the peculiar difference that in *Ec*AAH the Mn^2+^ occupies site II (See comparison in Fig. 2c,e). The IH, KynB and AAH families are not the only ones to have divergent metal site constellation, despite similar metal binding residues, the ureidoglycolate amidohydrolase from *Arabidopsis thaliana* (*At*UAH) has a binuclear Mn^2+^ site^62^ (PDB ID 4PXB), while the putative hydantoinases (PDB ID 5I4M, 5THW and 4WJB) and β-alanine synthase from *Saccharomyces kluyveri*^63^ (PDB ID 2VL1) have binuclear Zn^2+^ sites, the L-N-carbamoylase from *Geobacillus stearothermophilus* has a mononuclear Co^2+^ site^64^ (PDB ID 3N5F). The model for hydrolysis presented here would *only* allow a Mn^2+^ in site I, and a Zn^2+^ would act as an inhibitor. In particular, according to previous simulations, Zn^2+^ is not able to bind both isatin and the water, disrupting the preorganization of the active site and preventing the initial nucleophilic attack^21^. Thus, it remains an open question whether the Mn^2+^ in site II also act as an inhibitor or merely adopt an alternative position in the structure of *Ec*AAH due to the crystal conditions. It is a reoccurring problem for metal dependent hydrolases that the majority of the information is collected from *in vitro* experiments and that conflicting conclusions are reached when it comes to metal specificity. As in the case of KynB were a binuclear Zn^2+^ is described in the structure^20^ and Zn^2+^ dependent activity is presented, however it still remains an open question whether this is the physiological metal, as in other systems such as *Ralstonia metallidurans* a 15 to 20‐fold activation of kynurenine formamidase was detected after treatment with Mn^2+^ ^65^.

The major difference between the Michaelis complexes in *La*IHA/B and zinc-β-lactamases is constituted by the binding mode of the substrate. β-lactam substrates, bind in the proximity of the metal(s), but their amide moiety do not participate directly to their coordination sphere(s) as is the case for the reacting cyclic amide in the IH. It is carboxylate groups of the carpapenem family, that later during the catalysis interact directly with the Zn^2+^ ion as proposed is for several beta lactamases^6–8^ including the more recent New Delhi metallo-β-lactamases^9,66,67^. In *La*IHA it is the oxygen of the electrophilic amide carbonyl on isatin that is directly coordinated to the catalytic Mn^2+^ ion (Fig. 3). The enzyme kinetics analyses, accompanying structural determination of *La*IHA and *Rs*IHA, present striking evidence that *Rs*IHA, *La*IHA, and *La*IHB are true orthologues enzymes. These data support the claim in Bjerregaard-Andersen *et al*.^17^ that isatin metabolism is present in bacteria native to the human gut and strengthen the hypothesis that the molecule isatin may be a signalling molecule that links the gut-brain axis under conditions such as stress^68,69^.

## Electronic supplementary material

Supplementary Information

Supplementary Video
