# Supplementary material for: A fundamental catalytic difference between zinc and manganese dependent enzymes revealed in a bacterial isatin hydrolase
Source: Sci Rep. 2018 Aug 30;8:13104. doi: 10.1038/s41598-018-31259-y (PMC6117287; doi:10.1038/s41598-018-31259-y)
Supplement: Supplementary file 2 — Supplementary Video [file 41598_2018_31259_MOESM2_ESM.pdf]

Supplementary Material for:

A fundamental catalytic difference between Zinc and Manganese dependent enzymes revealed in a bacterial Isatin hydrolase

Theis Sommer[1,*], Kaare Bjerregaard-Andersen[2,*], Lalita Uribe[5,6*], Michael Etzerodt[4], Gregor Diezemann[5], Jürgen Gauss[5], Michele Cascella[2,7], J. Preben Morth[1, 3]

[1] Norwegian Center for Molecular Medicine, Nordic EMBL Partnership University of Oslo, Gaustadalléen 21, 0349 Oslo, Norway

[2] Department of Chemistry, University of Oslo, Sem Sælands vei 26, 0371, Oslo, Norway

[3] Institute for Experimental Medical Research, Oslo University Hospital, N-0424 Oslo, Norway

[4] Department of Molecular Biology and Genetics, Aarhus University, Gustav Wieds vej 10C, DK-8000 Aarhus, Denmark

[5] Institut für Physikalische Chemie, Johannes-Gutenberg Universität Mainz, Duesbergweg 10-14, 55128 Mainz, Germany

[6] Graduate School Materials Science in Mainz, Staudinger Weg 9, 55128, Germany

[7] Hylleraas Centre for Quantum Molecular Sciences, University of Oslo, Sem Saelands vei 26, 0371, Oslo, Norway

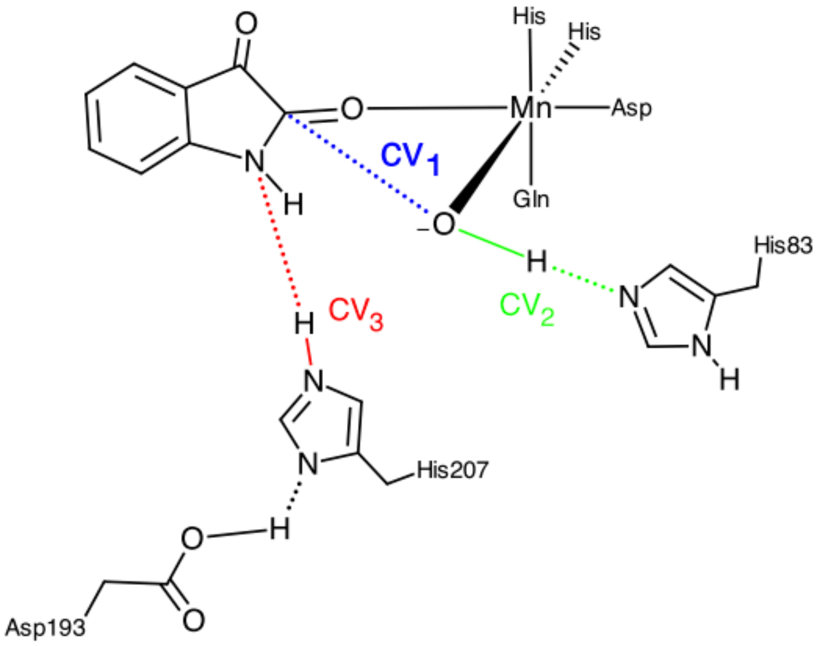

Supplementary Figure S1, Graphical view of the collective variables (CV_1-CV_3) defined in the metal binding pocket of *la*IHA, for the QM/MM - metadynamics simulations presented in the main text.

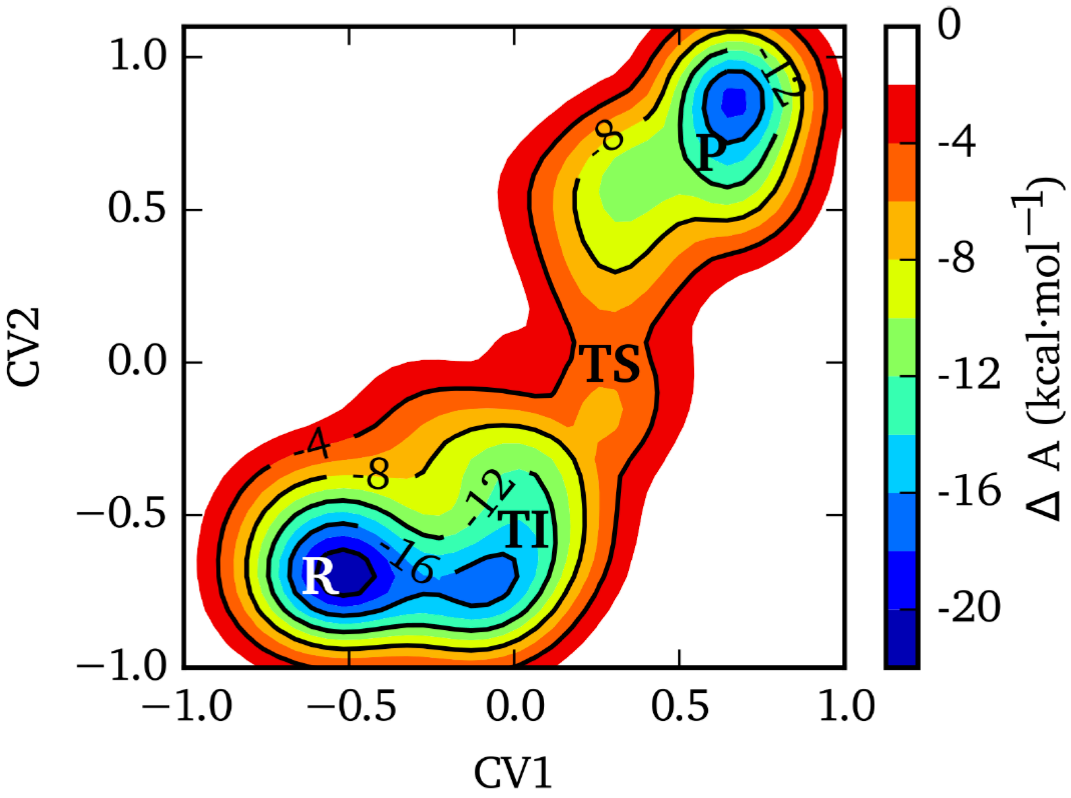

Supplementary Figure S*2,* Projection of the free energy surface over the CV1-CV2 coordinates. The letters in the plot indicate: (R) the reactant state, (TI) the tetrahedral intermediate, (TS) the transition state, and (P) the isatinate product.

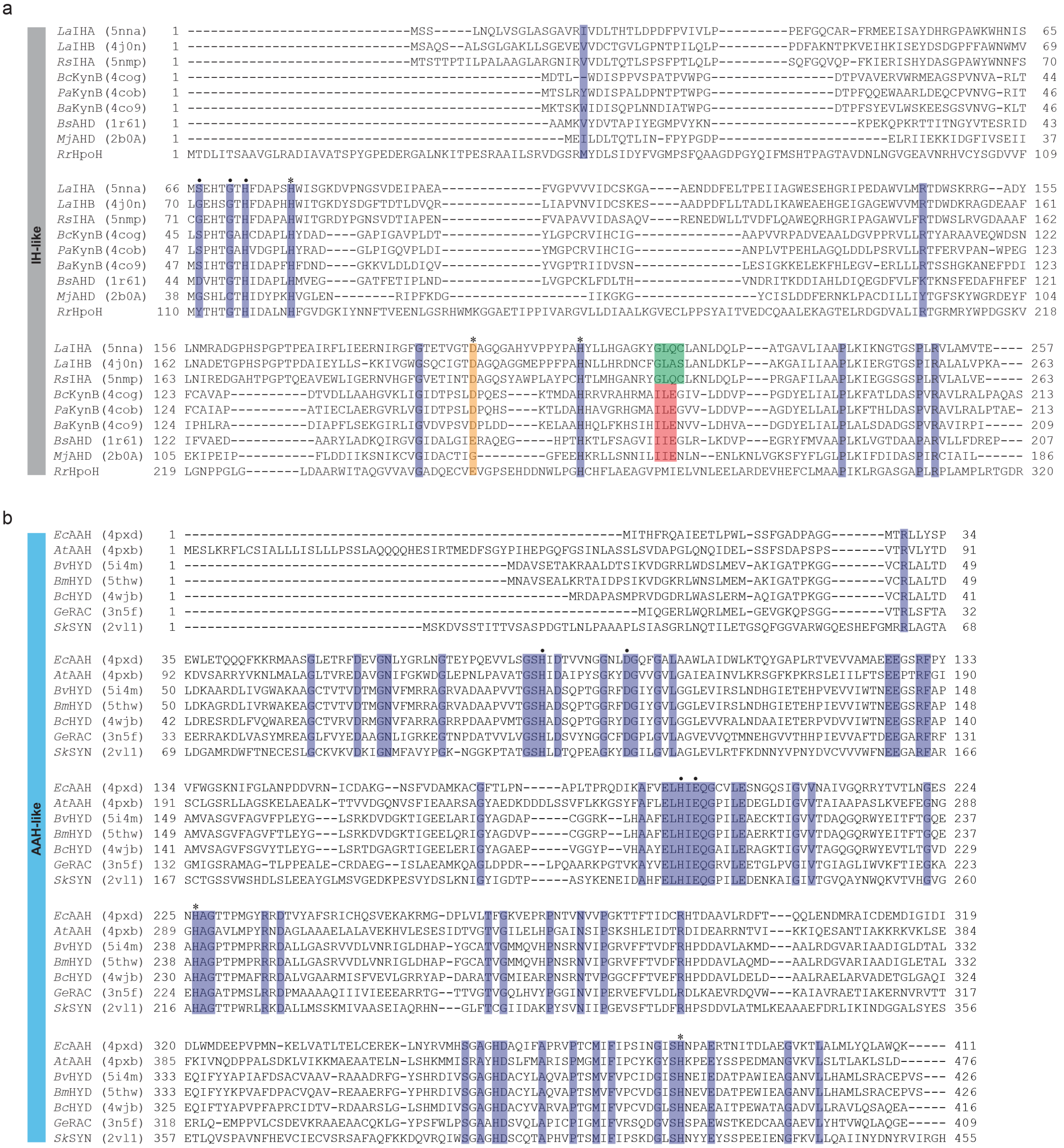

Supplementary Figure S3. Complete sequence alignments of partial alignment shown in Figure 2a. a) Isatin hydrolase homologues, b) Allantoate amidohydrolase homologues. Conserved residues are highlighted in blue. Residues involved in metal coordination are indicated by a dot, residues presumed to be involved in proton transfer are indicated with an asterisk. The key GLQC/GLAS motif of IHA and IHB, indicating isatin hydrolase activity, is highlighted in green, with a similarly conserved motif in KynB homologues highlighted in red.

Supplementary Table 1. X-ray crystallographic data collection and refinement statistics for IHA

	*La*IHA:Benzylbenzoate[1]	*Rs*IHA[1]	*La*IHA:isatinate[1]
PDB ID	5NNA	5NMP	5NNB
**Data collection**	Diamond, I02-P1	ESRF, ID30B	PETRA III
Space group	P 1	P 32 2 1	P 1
Wavelength (Å)	1.0004	0.9762	1.033
Cell dimensions a, b, c (Å)	37.7, 70.7, 92.9	164.8, 164.8, 275.9	38.4, 69.9, 93.5
α, β, γ (°)	108.2, 95.9, 101.5	90.0, 90.0, 120.0	108.2, 95.3, 103.8
Resolution (Å)[2]	86.86 – 1.50 (1.54 – 1.50)	99.2 - 2.65 (2.65-2.72)	19.5 - 1.79 (1.9 – 1.79)
R_{merge}[2]	0.073 (0.421)	0.093 (1.093)	0.052 (0.496)
$I / \sigma I$[2]	10.1 (2.0)	15.6 (1.5)	7.99 (1.0)
Completeness (%)[2]	96.1 (93.7)	99.8 (99.4)	94.05 (94.6)
Redundancy	3.4	6.9	1.8
CC½	0.995(0.937)	0.997 (0.658)	0.996 (0.816)
**Model refinement**			
No. observed reflections[2]	461622 (32325)	867299 (64074)	263115 (41566)
Unique[2]	135172(9712)	125897 (9226)	141099 (22382)
R_{work}	0.149	0.152	0.1826
R_{free}	0.180	0.196	0.2332
No. atoms	9195	20605	8307
Protein	8065	20300	784
Ligand/ion	106	10	48
Water	1024	295	419
B-factors (Å^2)			
Protein	12.2	68.8	40.5
Water	26.9	57.0	54.0
Ligand/ion	21.1	58.6	41.5
Monomers (asymmetric unit)	4	10	4
R.m.s. deviations			
Bond lengths (Å)	0.006	0.008	0.007
Bond angles (°)	1.06	1.04	1.01
Ramachandran plot, residues in:			
Favored region (%)	98	96	98
Allowed regions (%)	1.6	3.5	1.6
Outlier region (%)	0.4	0.5	0.4

[1] *Complete diffraction data was obtained from one crystal*

[2] *Values in parentheses are for highest-resolution shell.*
